# The Combined Effect of Heat and Osmotic Stress on Suberization of *Arabidopsis* Roots

**DOI:** 10.3390/cells11152341

**Published:** 2022-07-29

**Authors:** Ana Rita Leal, Joana Belo, Tom Beeckman, Pedro M. Barros, M. Margarida Oliveira

**Affiliations:** 1 Instituto de Tecnologia Química e Biológica António Xavier, Universidade Nova de Lisboa (ITQB NOVA), GPlantS, Av. da República, 2780-157 Oeiras, Portugal; ana.leal@itqb.unl.pt (A.R.L.); jbelo@itqb.unl.pt (J.B.); 2 Department of Plant Biotechnology and Bioinformatics, Ghent University, Technologiepark 71, 9052 Ghent, Belgium; tom.beeckman@psb.vib-ugent.be; 3 Center for Plant Systems Biology, VIB, Technologiepark 71, 9052 Ghent, Belgium

**Keywords:** abiotic stress, ATR-FTIR, plant cell wall, secondary development, suberin

## Abstract

The simultaneous occurrence of heat stress and drought is becoming more regular as a consequence of climate change, causing extensive agricultural losses. The application of either heat or osmotic stress increase cell-wall suberization in different tissues, which may play a role in improving plant resilience. In this work, we studied how the suberization process is affected by the combination of drought and heat stress by following the expression of suberin biosynthesis genes, cell-wall suberization and the chemical composition in *Arabidopsis* roots. The *Arabidopsis* plants used in this study were at the onset of secondary root development. At this point, one can observe a developmental gradient in the main root, with primary development closer to the root tip and secondary development, confirmed by the suberized phellem, closer to the shoot. Remarkably, we found a differential response depending on the root zone. The combination of drought and heat stress increased cell wall suberization in main root segments undergoing secondary development and in lateral roots (LRs), while the main root zone, at primary development stage, was not particularly affected. We also found differences in the overall chemical composition of the cell walls in both root zones in response to combined stress. The data gathered showed that, under combined drought and heat stress, *Arabidopsis* roots undergo differential cell wall remodeling depending on developmental stage, with modifications in the biosynthesis and/or assembly of major cell wall components.

## 1. Introduction

Extreme climate events related to climate change often lead to the simultaneous occurrence of different abiotic stresses, which severely affect crop growth and yield [1]. In particular, the combination of heat and drought stress is described to cause extensive agricultural losses [2]. However, despite the frequent occurrence of the combined stress episodes under field conditions, most studies involving both model and crop plants have mostly focused on independent heat or drought stress responses. Generally, heat and drought have shown some opposite effects when applied alone (under controlled conditions). In response to heat, plants open the stomata to increase transpiration and cool the leaf surface, while the opposite is observed during drought treatments, with stomata closing to prevent water loss [3,4]. In adaptation to drought, the increased root growth may be also observed as a strategy to optimize water uptake [5]. While studies focusing on plant response to drought and heat stress (applied independently) have provided important insights on the plants molecular and physiological adaptations, these may disregard interactions between stresses. Still, several transcriptomic studies of combined stresses have been performed [4,6,7,8], revealing that only a small number of the genes expressed in single stresses also show up in combined stresses [4,6,7,8]. Despite these obvious differences, it has been reported that heat and drought, alone or in combination, induce similar results, affecting flowering time, seed development and final yield [6,9,10].

Several studies have shown that environmental and/or growth conditions can modulate root suberization at endodermis and/or periderm [11,12,13,14,15,16]. Generally, abiotic stresses cause increased suberization in several plant species and tissues [17,18,19,20,21,22,23]. In barley modern cultivars, osmotic stress induced cell wall suberization of endodermis [24,25], but this response is not as evident in the wild accessions [21]. Enhanced suberization in modern cultivars significantly reduces radial water transport, whereas wild accession can keep the water transport constant, in combination with other adaptation mechanisms [21]. Increased suberin deposition in roots, often with upregulation of suberin biosynthesis genes, was also found in response to drought in grapevine [26], rice [17] and *Arabidopsis* [19,27]. Plasticity in root suberization may act to reduce water leakage from xylem and/or minimize excessive salt penetration into the stele and accumulation at toxic concentrations. The impact of heat stress in suberization was first characterized on potato skin. Heat-stressed potato tubers develop multiple suberized and denser skin layers, with higher suberization and increased expression of suberin biosynthesis genes [18,28]. The increased suberization improves potato thermal-insulation, reducing water loss from the tuber flesh.

Interestingly, the phytohormone abscisic acid (ABA), the main signaling hormone detected in heat and drought response [5,29], is also described as the main transcriptional activator of suberin biosynthesis genes [30,31]. By mimicking the abiotic stress stimulus [29], ABA application induces suberin biosynthesis genes, endodermis suberization as well as ectopic deposition of suberin in cells that normally do not suberize [30,32].

In this work, we aimed to clarify how *Arabidopsis* root suberization is modulated by combined drought and heat stress by targeting the roots at the onset of secondary development. At this stage, the *Arabidopsis* main root displays a gradient of development, from primary (closer to root tip) to secondary growth (below the hypocotyl) [31,33], with two distinct suberized tissues: the endodermis, in the region of primary development, and the phellem (the outermost layer), in the region of secondary development. Suberized endodermis plays a role as transport barrier to the stele, while phellem is mostly a protective layer. Our study aimed to assess if changes in suberization in response to stress were global or tissue-specific. Using a combined approach, we followed the expression of suberin biosynthesis genes and changes in cell-wall composition, and found that the imposition of combined heat and osmotic stress produced a differential effect on suberization depending on the root developmental stage.

## 2. Materials and Methods

### 2.1. Plant Material

*Arabidopsis thaliana* ecotype Columbia (Col-0) was used for gene expression and chemical analysis. The transgenic line pGPAT5::mCITRINE-SYP122, previously reported by Barberon et al. [30] was used for fluorescence microscopy.

### 2.2. Plant Growth Conditions and Combined Stress Treatment

For in vitro growth assays, seeds were surface sterilized with 70% (*v*/*v*) ethanol for 5 min, 20% (*v*/*v*) bleach for 5 min and rinsed five times with sterile water. Seeds were further sown on Petri dishes containing sterile half-strength Murashige and Skoog (1/2 MS) medium [0.5 × MS salts, 1% sucrose, 0.5 g/L 2-(N-morpholino) ethanesulfonic acid MES, 0.1 g/L Myo-Inositol and 1% *w*/*v* agar, pH 5.7] and stratified at 4 °C for 3 days in the dark. Seeds were germinated in the vertical position in a growth chamber at 21 °C under continuous light (100 µmol m^−2^ s^−1^).

Polyethylene glycol (PEG)-infused medium was prepared on plates by overlaying an equal volume of 40% PEG 6000 solution (dissolved in 1/2 MS and re-adjusting the pH to 5.7) on a same volume of solid 1/2 MS medium. The agar medium and PEG solution was then allowed to equilibrate for at least 12 h and the excess liquid solution was further removed, resulting in 1/2 MS plates supplemented ~20% PEG 6000 [34,35].

For the combined heat and osmotic stress treatment, plants were transferred to PEG-infused plates 8 days after germination (DAG), and grown at 32 °C for 24 h (continuous light, 100 µmoL m^−2^ s^−1^). For control treatments, 8 DAG plants were transferred to new 1/2 MS plates, and grown at 21 °C with the above-indicated light conditions. Control plants were taken for analyses after exactly the same number of hours in the new medium as the hours of stress imposed on the test plants (0–24 h). Similarly, for abscisic acid (ABA) treatment, 8 DAG plants were transferred to 1/2 MS plates supplemented with 1 µM ABA and grown in parallel with control plants (transferred to same medium without ABA) at 21 °C and light as above.

### 2.3. Microscopy

Confocal laser scanning microscopy imaging was performed either with a Leica SP5 or a Zeiss LSM 710. Excitation and detection windows were set as follows: Propidium iodide: Ex: 514 nm and Em: 650–700; mCITRINE: Ex: 488 nm and Em:500–550 nm; Fluorol Yellow: Ex: 488 nm and Em: 500–550 nm. For Fluorol Yellow (FY) staining [36], plants were incubated in a freshly prepared solution of 0.01% (*w*/*v*) FY (Santa Cruz Biotechnologies) in lactic acid (80%) at 70 °C for 30 min. The samples were washed (3 × 5 min) in water and counter-stained with aniline blue (0.5% *w*/*v*, in water) at room temperature in darkness for 30 min. Afterwards, plants were again washed (3 × 10 min) in water and mounted on 50% glycerol. For propidium iodide (PI) staining, fresh plants were incubated in a fresh solution of 15 mM PI for 5 min, rinsed twice in water and mounted in 50% glycerol. For epi-fluorescence imaging, a Leica DM6 B Upright Microscope equipped with a L5 filter cube was used.

### 2.4. Gene Expression Analysis

Root material was collected before treatment (0 h) and 1 h, 2 h, 3 h, 4 h, 6 h and 24 h after each treatment. Roots were segmented for sampling two different zones: secondary development zone, containing the region undergoing secondary development together with any formed lateral roots (1.5 cm from hypocotyl); and primary development zone, from root tip until the first visible lateral root (Supplementary Figure S1). The harvested root segments were immediately frozen in liquid nitrogen, with each collection point corresponding to a pool of 90–100 plants. Total RNA was extracted using the Direct-zol RNA kit (Zymo research). On column DNAse Itreatment was performed according to the manufacturers’ instructions. For quantitative PCR analysis, first strand cDNA synthesis was performed using 1 μg total RNA with an oligo-dT primer using Transcriptor High Fidelity cDNA Synthesis Kit (Roche), according to the manufacturer’s instructions. The cDNA was diluted and used as template for amplification by qPCR using gene-specific primers (listed on Supplementary Table S1). Ubiquitin-conjugating enzyme E2 (*UBQ21*, At5g25760) was used as internal control to calculate relative expression of the selected target genes, given its stable expression in the tested time points. Real Time qPCR was conducted in a Lightcycler 480 (Roche), using Lightcycler 480 Master I Mix (Roche). Amplification reactions were performed in triplicate for each cDNA sample. Target transcript abundance was calculated according to Pfaffl [37].

### 2.5. Attenuated Total Reflection Fourier Transform Infrared Spectroscopy (ATR-FTIR)

For ATR-FTIR analysis, roots were collected from primary and secondary development zones (as described above for RNA extraction, Supplementary Figure S1) for control and combined heat and osmotic stress, 24 h after treatment. Plant material was immediately washed with ice cold water for 5×, to remove growth media traces. Plant material was then flash frozen in liquid nitrogen, lyophilized and powdered prior to the analysis. A solid PEG6000 sample was used to identify possible PEG-specific contaminating peaks in the root samples. ATR-FTIR spectra were collected on a Bruker IFS66/S FTIR spectrometer (Bruker Daltonics, Billerica, MA, USA) using a single reflection ATR cell (DuraDisk, equipped with a diamond crystal). Data were recorded using OPUS v5.0 software, at room temperature, in the range of 4000–600 cm^−1^, by accumulating 256 scans with a resolution of 2 cm^−1^. Five replicate spectra were collected for each sample to evaluate reproducibility.

## 3. Results

### 3.1. Suberization Pattern of Arabidopsis Roots Changes during Combined Heat and Osmotic Stress

Combination of heat and osmotic stress (H+O) was imposed to *Arabidopsis* plants at 8 DAG, on the onset of root secondary development [31]. In order to evaluate the effect of combined stress on root suberization, we performed a morphological and spatio-temporal characterization of root suberization, targeting the expression of the suberin biosynthesis marker *Glycerol-3-phosphate acyltransferase 5-GPAT5* (pGPAT5:mCITRINE-SYP122 [30], as well as cell-wall suberization, using FY staining [36]).

**Figure 1:**
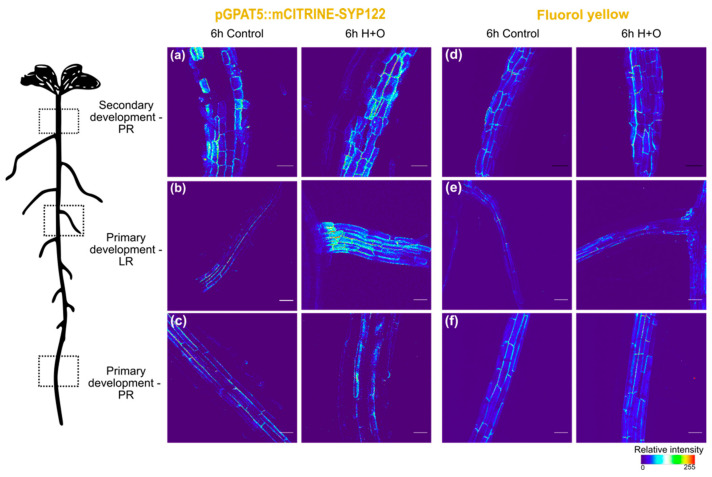
Effect of combined heat and osmotic stress on cell wall suberization of *Arabidopsis* primary root (PR), at secondary (**a**,**d**) and primary (**c**,**f**) development stages, and lateral roots (LR; **b**,**e**). Expression of suberin biosynthetic gene *GPAT5* was detected after 6 h of control and combined heat and osmotic stress treatments using pGPAT5::mCITRINE-SYP122 line (**a**–**c**). FY staining was used in col-0 *Arabidopsis* to detect cell wall suberization after 6 h of control and combined heat and osmotic stress treatments (**d**–**f**). The 3D maximum projection figures were obtained by confocal laser scanning with Z-stack images. Pixel values were converted in a thermal look-up-table. Scale bar-50 µm. For the corresponding bright field images, please refer to the Supplementary Figure S2.

After 6 h of H+O, *GPAT5* expression was detected in endodermis and phellem cells of the main root, with similar marker expression intensity as in control (Figure 1a,c and Supplementary Figure S1a,c). Furthermore, the FY staining showed similar fluorescence intensities in control as well as in treated plants (Figure 1d,f and Supplementary Figure S2d,f). In turn, in LRs *GPAT5* expression was detected in cortex cells of plants exposed to the combined stress (Figure 1b), while in control it was found only in the endodermis.

This ectopic *GPAT5* expression pattern was detected in 66% of total emerged LRs in the H+O stress, compared with only 5% of ectopic marker expression detected in LRs of control treated plants (Supplementary Figure S3). However, at this time point, ectopic suberin deposition was not detected by FY staining in LRs (Figure 1e).

**Figure 2:**
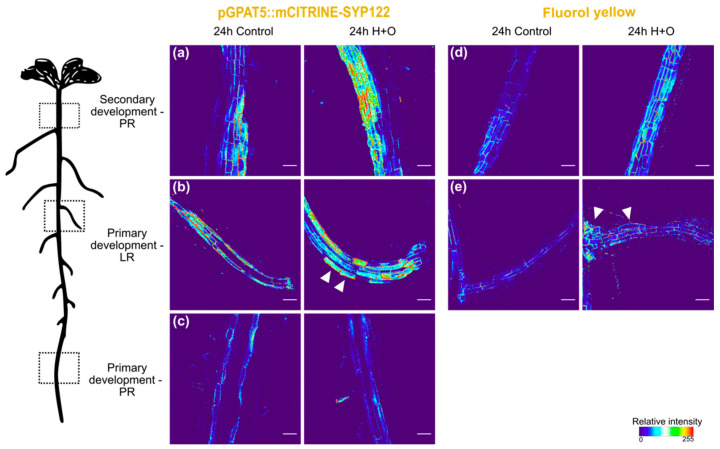
Effect of combined heat and osmotic stress on cell wall suberization of *Arabidopsis* primary root (PR), at secondary (**a**,**d**) and primary (**c**) development stages, and lateral roots (LR; **b**,**e**). Expression of suberin biosynthetic gene *GPAT5* was detected after 24 h of control and combined heat and osmotic stress treatments using the promoter line pGPAT5::mCITRINE-SYP122 (**a**–**c**). FY staining was used in col-0 *Arabidopsis* to detect suberin after 24 h of control and combined heat and osmotic stress treatments (**d**,**e**). Arrowheads indicate *GPAT5* expression and FY staining in cortex cells of LRs. The 3D maximum projection figures were obtained by confocal laser scanning with Z-stack images. Pixel values were converted in a thermal look-up-table. Scale bar-50 µm.

After 24 h, *GPAT5* promoter activity was increased in phellem cells of stress-treated plants (Figure 2a). In parallel, FY staining fluorescence was also stronger in this region, as compared with roots under control conditions (Figure 2d). In LRs, *GPAT5* ectopic expression is again detected after 24 h of H+O treatment (Figure 2b, arrowheads; Supplementary Figure S3). In addition, suberin was detected by FY staining in LR cortex cells (Figure 2e, arrowheads). In young main root, as in the previous time point, no differences were detected with *GPAT5* expression (Figure 2c), as well as for FY staining, which was only detected in endodermis, similarly to control treatment.

These results suggest that the root suberization process, including biosynthesis and deposition of suberin in cell walls is induced within 24 h after the imposition of the H+O stress treatment, particularly in phellem and cortex cells of young lateral roots.

**Figure 3:**
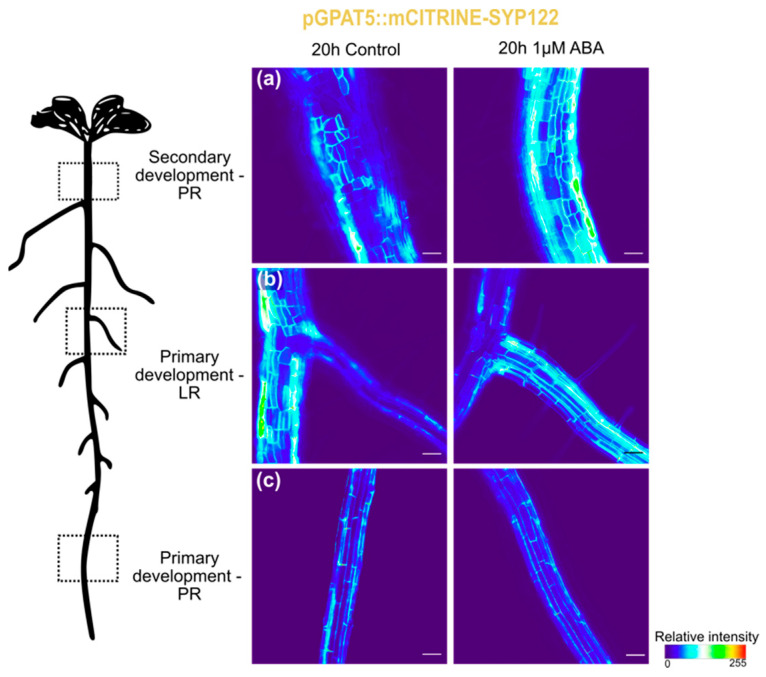
Expression of *GPAT5* (pGPAT5::mCITRINE-SYP122 line) in the primary root (PR), at secondary (**a**) and primary (**c**) development stages, and lateral roots (LR; (**b**)), under control conditions and 20 h after ABA treatment. Pixel values were converted in a thermal look-up-table. Scale bar represents 50 µm.

To elucidate if the signaling pathway mediating suberization on *Arabidopsis* roots under the H+O treatment was ABA-dependent, we further tested the external application of ABA (1 µM). After 20 h of ABA treatment, *GPAT5* marker was detected in cortical and epidermis cells of LR (Figure 3b), and, mostly, no differences were detected in main root, either at primary or secondary growth zones, when compared with control treatments (Figure 3a,c). This result suggests ABA as candidate regulator of ectopic *GPAT5* expression in LRs in response to combined heat and osmotic stress.

### 3.2. Differential Gene Expression in Suberization Induced under Combined Heat and Osmotic Stress

To get a wider view over the molecular pathways mediating suberin deposition in roots in response to H+O stress, we further evaluated the expression of several genes with demonstrated involvement in suberization. Considering the distinct responses detected between root zones, we sampled the root in two distinct segments (Supplementary Figure S1). Zone A, which included secondary developed root zone and LRs, and Zone B, which was collected from main root tip to the first emerged LR. The targeted genes included *GPAT5* [38], *Aliphatic Suberin Feruloyl Transferase-ASFT* [39] and *ATP-Binding Cassette G6-ABCG6* [40], which are involved in different steps of suberin biosynthesis and deposition, and *MYB41* transcription factor, reported as a regulator of suberization process in response to osmotic, ABA and salt stress [32]. In addition, an *FK506-binding protein-ROF2* and *Responsive to dehydration 22-RD22* were selected as marker genes for heat and osmotic stresses, respectively. *Casparian strip membrane protein 1-CASP1* expression was also assessed to determine if Casparian strip was affected by the imposed stress.

**Figure 4:**
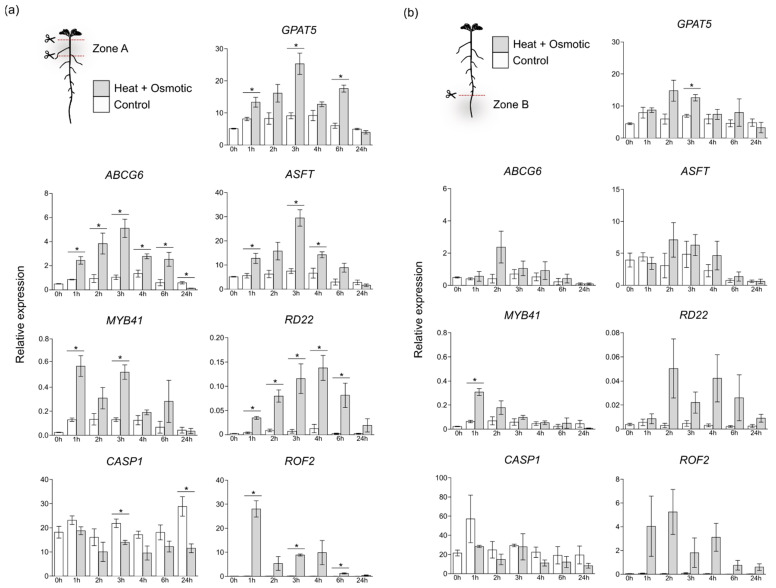
Expression of selected genes involved in suberization and/or response to stress, in roots of *Arabidopsis* plants submitted to combined heat and osmotic stress (gray bars) as compared to control (white bars) treatments. Root zones A (**a**) and B (**b**) were analyzed individually at selected time points from 0 h (before stress imposition) to 24 h after stress initiation. Gene expression was analyzed by RT-qPCR and normalized with the housekeeping gene *UBQ21*. Mean expression ± standard deviation from 3 independent experiments is represented for each time point. Asterisks (*) represent significant differences (*p* < 0.05, unpaired *t*-test) between control and combined heat and osmotic stress for a given time point.

In zone A (Figure 4a), genes involved directly in the suberization process-*GPAT5*, *ABCG6* and *ASFT*-showed a similar expression pattern, being upregulated shortly after 1 h of stress imposition, and reaching a peak of expression at 3 h (3 to 5-fold upregulation, compared to control). After this time-point, gene expression decreased in combined stress, reaching the levels detected for control treatment, at 24 h. In turn, *MYB41* expression peaks shortly at 1 h after combined stress application (>3-fold upregulation, compared to control), and gradually decreased after 3 h. Interestingly, *ROF2* expression was also highly upregulated 1 h after stress initiation, decreasing afterwards and reaching basal levels at 24 h in stress. The osmotic stress-specific *RD22* gene showed a stepwise increase in expression in the first hours after stress, reaching a peak only after 4 h, and decreasing in the following time-points analyzed. In contrast, *CASP1* expression was downregulated in response to combined H+O stress, showing reduced levels of expression along the 24 h treatment.

In contrast to zone A, the impact of combined stress in the expression of suberin-related genes was less evident in zone B (Figure 4b). While the expression of *GPAT5*, *ASFT* and *ABCG6* was also induced by combined stress, at specific time points, the mean expression differences to control conditions were mostly not significant. Nevertheless, an expression peak was detected for these genes at 2 h after combined stress imposition. An upregulation of *MYB41* expression was also detected in this root zone 1 h after treatment, returning to levels closer to control after 2 h. Equally, expression of *ROF2* and *RD22* was also induced by stress, but this response was again less pronounced than that observed for root zone A (Figure 4a,b). In turn, *CASP1* expression did not suffer significant alterations in this region of the root after stress imposition.

Overall, these results support a differential impact of combined H+O stress depending on the targeted root zones. As observed by fluorescence microscopy, we found a stronger induction in the expression of genes related to suberin biosynthesis in the mature root zones, containing LRs and suberized phellem, in comparison to the primary root undergoing primary development.

### 3.3. Combined Heat and Osmotic Stress Changes Root Cell Wall Composition

**Figure 5:**
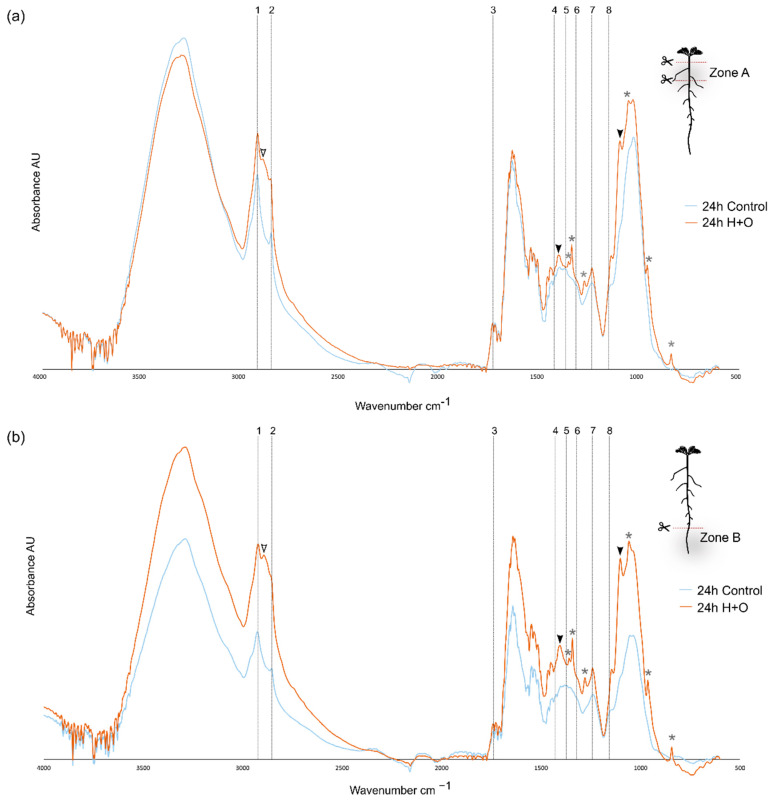
ATR-FTIR spectra determined for *Arabidopsis* root zones A (**a**) and B (**b**) at 24 h after application of combined heat and osmotic stress (H+O) and control. Vertical dashed lines highlight major peaks assigned for suberin groups: 1 and 2—long aliphatic chains of suberin (2921, 2852 cm^−1^); 3, 7 and 8—carboxyl and ester groups (1737, 1242, and 1158 cm^−1^); 4—phenolic compounds (1430 cm^−1^); 5 and 6—wax or suberin-like aliphatic components (1318 and 1372 cm^−1^). Arrowheads indicate novel peaks detected in response to combined stress. Asterisks indicate strong peaks resulting from some contamination with PEG6000 used in osmotic stress imposition (see Supplementary Figure S5).

The impact of combined H+O in global cell wall composition in the different zones of the root was further assessed by ATR-FTIR (Figure 5). This is a fast procedure that provides information on cell wall polymers and functional groups, with minimal sample processing. The fundamental vibration modes of molecular functional groups produce characteristic spectral absorption features that can serve to ‘fingerprint’ many cell wall compounds [41]. For strongly suberized cells, the ATR-FTIR spectra is predicted to peak at specific wavenumbers, generating a fingerprint representing typical suberin monomer groups, namely: long aliphatic chains (2921, 2852 cm^−1^), carboxyl and ester groups (1737, 1242, and 1158 cm^−1^), phenolic compounds (1430 cm^−1^) and wax or suberin-like aliphatic components (1318 and 1372 cm^−1^) [42,43,44] (Supplementary Table S2).

ATR-FTIR spectra were obtained for the root zones A and B from plants subjected to 24 h of combined H+O and for control treatments (Figure 5 and Supplementary Figure S1). Under control conditions, the ATR-FTIR spectra obtained for the two different developmental zones (Zone A and Zone B) were similar, and several suberin-related peaks could be identified, representing the aliphatic chains and carboxyl and ester groups (Supplementary Figure S4, dashed lines 1–3,7–8). The main difference between spectra obtained for zones A and B was found between 1030 and 1053 cm^−1^ (Supplementary Figure S4, arrowheads). Strong bands between 1032 cm^−1^ and 1055 cm^−1^ are assigned to the C–O stretching of primary and secondary alcohols [45,46] (Supplementary Table S2), associated with diverse polysaccharides, such as cellulose, hemicellulose and pectin molecules [47]. In Zone B, a plateau was detected in this region, while a peak at 1035 cm^−1^ was found for zone A sample (Supplementary Figure S4, arrowheads). These results suggest that in control conditions, root zone B has an equivalent composition in primary and secondary alcohols from diverse polysaccharides, whereas in root zone A, the primary alcohols are prevalent.

The ATR-FTIR general profile obtained for root samples after 24 h of combined H+O stress was similar to control, in what concerns the distribution of absorbance peaks attributed to suberin monomer groups, for both root zones analyzed (Figure 5, dashed lines). However, other specific changes were observed in samples of plants submitted to stress, regardless of the root zone. More specifically, new absorbance peaks (Figure 5, arrowheads) were found, namely at 2889, 1407 and 1097 cm^−1^. Absorbance at 2889 cm^−1^ in ATR-FTIR spectra is assigned to cellulose molecules (C-H stretching vibrations) [48] (Figure 5, white filled arrowheads), while 1407 and 1097 cm^−1^ are both assigned to pectin [49,50] (Figure 5, black filled arrowheads) (Supplementary Table S2). The additional absorbance peaks identified in H+O groups (Figure 5, asterisks) are likely due to the presence of residual PEG (used to mimic drought), given the overlap with the spectra obtained for this compound alone (Supplementary Figure S5, asterisks).

The global changes detected by the ATR-FTIR spectra suggests that the combined heat and osmotic stress induced changes in the cell wall load of cellulose and pectin.

## 4. Discussion

Suberization is being regarded as an interesting trait to improve plant response to abiotic stress. Several studies have shown that changes in root suberin deposition can affect the way plants cope with the applied stresses. Heat and drought showed different effects on plants when applied alone [3,4], and opposite effects were described specifically for root growth [5]. In contrast, heat and drought alone have shown similar effects concerning suberization, with increasing suberization being detected in several plant species and organs when individual stresses are applied [17,19,26,27]. Thus, it is of great importance to study the effect of abiotic stress in suberization, considering the root anatomy and developmental stage, to better understand the influence of suberized barriers in adaptation to stress.

In this work, we used 8 DAG *Arabidopsis* to study the influence of a combination of heat and osmotic stress on root suberization. At 8 DAG, *Arabidopsis* root system is already complex, with secondary development present in a small portion of the upper part of the main root, with suberized phellem as the outermost layer [31], and below this region, in primary development, the main root has a layer of suberized endodermis around the stele. In addition, LRs are already present, showing equivalent morphology of younger roots, both undergoing primary development.

Using plants undergoing secondary growth, we could detect a distinct response to the combined heat and osmotic stress in the different developmental zones of the root system. In the endodermal cells of the main root, no significant differences were observed in response to stress, regarding expression of suberin biosynthesis genes or suberin deposition. Contrastingly, in the mature part of the root, phellem cells showed increased *GPAT5* expression, as well as a stronger FY staining, after the combined heat and osmotic treatment (Figure 1 and Figure 2). On the other hand, the suberization pattern of LRs was drastically altered upon combined stress treatment, with the activation of *GPAT5* expression and suberin deposition being detected, not only in the endodermis cells, but also in cortex and epidermal cells, which do not undergo suberization under control conditions (Figure 1 and Figure 2).

In our study, the expression of suberin-related genes and the overall suberization pattern was almost undisturbed by the combined stress in the main root zone undergoing primary development (zone B), as compared with the region under secondary development (zone A) (Figure 4). It is noteworthy that the increased expression levels of the targeted genes observed in zone A is likely influenced by the presence of LRs, since the results obtained by the histochemical analysis clearly showed a strong induction of suberization in LRs.

Interestingly, ABA treatment induced a similar response to combined heat and osmotic stress, with ectopic activation of *GPAT5* suberin marker in cortex and epidermis cells of LRs, whereas no obvious alteration was detected in the main root. Previous studies have demonstrated that ABA induces expression of suberin biosynthesis genes in tissues that are commonly suberized, but may also induce ectopic suberization in cells from other tissues [27,30,32,40,51]. More particularly, ABA treatment of 4 DAG *Arabidopsis*, induced the early onset of endodermis suberization, but also activated ectopic suberization in several cortex and epidermal cells of the main root [30]. ABA treatment also induced ectopic expression of the suberin synthesis regulator *MYB41* [32] namely in the root cortex and epidermis of 5 days-old *Arabidopsis* plants. Ectopic suberization or activation of suberin-related genes induced by ABA was only experimentally tested in young *Arabidopsis* roots, with no ongoing secondary development [30,32]. However, this ABA-induced suberization phenotype described in roots from younger plants is identical to the suberization phenotype that we described in LRs from 8 DAG plants after combined stress imposition, pointing for a role of ABA in this process. Future work aiming to clarify the role of ABA in suberization may gain from using ABA mutants. For instance, to uncover the ABA role in stress response, as well as in the normal phellem development, the mutants *aba2* (ABA biosynthesis) or *abi3*, *abi4* and *abi5* (ABA response), or even *abi1-1* (dominant-negative allele of the PP2C-type protein phosphatase ABI1), driven by a phellem-specific promoter, could be enlightening. On the other hand, the repression of the ABA signal after stress imposition [52,53] could clarify if the stress-induced suberization pattern observed in lateral roots is an ABA-specific response.

Using ATR-FTIR we obtained a preliminary understanding of other compositional changes in root cell walls in response do combined stress. ATR-FTIR has been extensively used to analyze and monitor cell wall changes occurring as a result of various processes, such as growth and development [44,54], genetic modifications [55] and responses to abiotic or biotic stresses [56]. In the analysis of the different root zones collected 24 h after stress imposition, no differences were detected in typical absorbance peaks attributed to suberin. Despite the increased suberization found in response to combined stress (as assessed by microscopy and gene expression analyses), the suberin monomeric relative composition is apparently unaltered. Nevertheless, changes in the ATR-FTIR spectra indicated other possible alterations in root cell walls induced by the combined stress treatment. For both root developmental zones analyzed, ATR-FTIR spectra showed new peaks in stress-treated roots that suggest a relative increase in cellulose and pectin levels (new bands at 2889 and 1407 and 1097 cm^−1^, respectively). It is widely described that plant cell walls are structurally organized in two separate networks of pectin and hemicellulose/cellulose [57]. Although this model is still generally accepted, increasing evidence shows interactions between these two networks and a more dominant role of pectin as part of the load-bearing cell wall structures [58]. In response to drought, increased amount of pectin and more ramified pectin backbones were proposed to form hydrogel capsules that could limit cell damage [59,60]. Furthermore, heat stress showed to modulate the degree of methylesterification/ramification of the pectins, by the induction of pectin methylesterase [61,62,63]. The identification of bands attributed to pectin in root samples exposed to combined stress may indicate not only a relative increase in pectin levels, but probably also in its side chains/ramifications. Further investigation on pectin methylesterase gene expression could provide additional support to this hypothesis. Regarding cellulose levels, a contrasting effect of different abiotic stresses has been described [64]. Mild osmotic or ionic stress caused a decrease in cellulose synthesis by rapid internalization of cellulose synthase enzymes (CesAs) from the plasma membrane into small subcellular compartments [65,66]. In turn, high temperatures alone caused an increase of CesA movement in the plasma membrane and a decrease in crystalline cellulose [67] suggesting an opposite relationship between the rate of cellulose synthesis and its crystallinity [68]. Additionally, several lines of evidence point for an increase in cellulose content when plants are exposed to heat stress [61,69,70]. Since ATR-FTIR analysis on non-extracted cell walls only provide a limited assessment on relative quantification of its component, further analysis may be conducted to monitor the cell wall alterations and elucidate specific changes in cell wall components, for example through mass spectrometry [71]. For example, mass spectrometry quantification of suberin monomers could be made using suberin deficient mutants, and more information could be gathered on the impact of stress on the suberization process. This would further allow identifying and quantifying suberin components in the different regions of the root, validating the suberization microscopy differences detected in stress treatments [72]. Transmission electron microscopy, could also help investigating cell wall modifications across different root developmental zones and stress-imposed treatment, specifically regarding cell wall thickening or modified suberin lamella features in different cell types [73,74].

The plant cell wall is critical to maintain cellular structural integrity, simultaneously providing resistance to internal hydrostatic pressures and flexibility to support cell division and expansion in tissue differentiation, as well as acting as environmental barrier protecting the cells in stress response. Alterations in cell wall pectin and cellulose components and structure upon combined stresses must also strongly influence the cell wall performance in its structural functions. Moreover, the observed expansion of suberization into the cortex and epidermis of LRs should profoundly impact the water and nutrient transport capacity of roots. More than detected increases in suberin, pectin and cellulose, our results support the idea that upon combined abiotic stress, the plant undergoes cell wall reorganization, with modified biosynthesis and/or assembly of major cell wall components, as also defended by Ezker et al. [75]. Further investigation of the overall alterations of the cell wall under combined heat and osmotic stress, including the structure and interconnection between pectin, suberin and cellulose moieties, may help to understand how these plant barriers contribute for the overall plant stress response. Additionally, as explained above, the use of ABA mutants can be helpful to clarify the ABA specific role in stress response and suberization patterns.

## Supplementary Materials

The following supporting information can be downloaded at: https://www.mdpi.com/2073-4409/11/15/2341/s1, Figure S1: Schematic representation of the sampling performed in *Arabidopsis* roots; Figure S2: Effect of combined heat and osmotic stress on the suberization pattern of secondary and primary development in lateral (LR) and primary root (PR) of *Arabidopsis*; Figure S3: Percentage of lateral roots (LR) with ectopic pGPAT5::mCITRINE-SYP122 (GPAT5) expression after 6 h and 24 h of combined heat and osmotic stress; Figure S4: ATR-FTIR spectra of the *Arabidopsis* secondary and primary developmental zone of root, collected 24 h undercontrol condition; Figure S5: ATR-FTIR spectra of solid PEG 6000; Table S1: Primers used in this work; Table S2: Summary of main peaks obtained by ATR-FTIR that are assignment to cell wall components.
