# Supplementary material for: The Combined Effect of Heat and Osmotic Stress on Suberization of Arabidopsis Roots
Source: Cells. 2022 Jul 29;11(15):2341. doi: 10.3390/cells11152341 (PMC9367520; doi:10.3390/cells11152341)
Supplement: Supplementary file 1 [file cells-11-02341-s001.zip › cells-1822287-supplementary.pdf]

The combined effect of heat and osmotic stress on suberization of Arabidopsis roots

Ana Rita Leal [1, 2, 3], Joana Belo[1], Tom Beeckman[2, 3], Pedro Barros[1*] and M. Margarida Oliveira[1*]

[1] Instituto de Tecnologia Química e Biológica António Xavier, Universidade Nova de Lisboa (ITQB NOVA), GPlantS, Av. da República, 2780-157 Oeiras, Portugal;

[2] Ghent University, Department of Plant Biotechnology and Bioinformatics, Technologiepark 71, 9052 Ghent, Belgium

[3] VIB Center for Plant Systems Biology, Technologiepark 71, 9052 Ghent, Belgium

Supplemental material

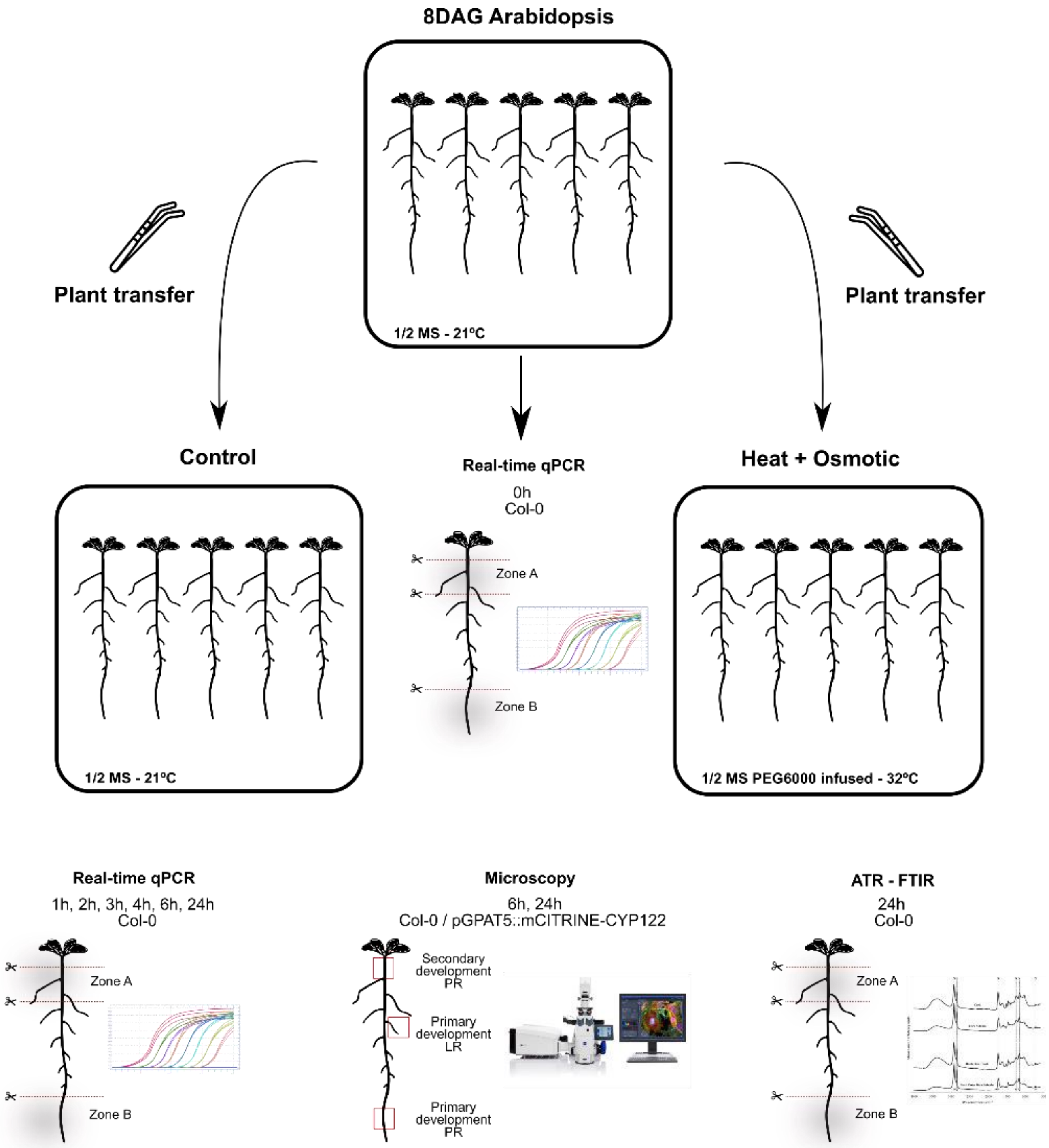

Figure S1 Schematic representation of the experimental set up and sampling performed in Arabidopsis roots. Arabidopsis plants were grown until 8 DAG in ½ MS medium, at 21°C. For the combined heat and osmotic stress, plants were transferred to ½ MS medium PEG 6000 infused plates and grown at 32°C. As control, plants were transferred to fresh ½ MS plates. For Real-time qPCR, Col-0 roots were segmented in two different zones: Zone A - secondary developmental zone, containing the main root undergoing secondary development and emerged lateral roots (±1.5 cm from hypocotyl); and Zone B - primary development zone, extending from the root tip until the first visible lateral root. Col-0 and pGPAT5::mCITRINE-CYP122 plants were used for fluorescence microscopy and observations were performed in the primary root (PR), at secondary and primary development stages, and lateral roots (LR). For ATR-FTIR analysis, Col-0 plants were collected and segmented in Zones A and B, as previously described for the Real-time qPCR.

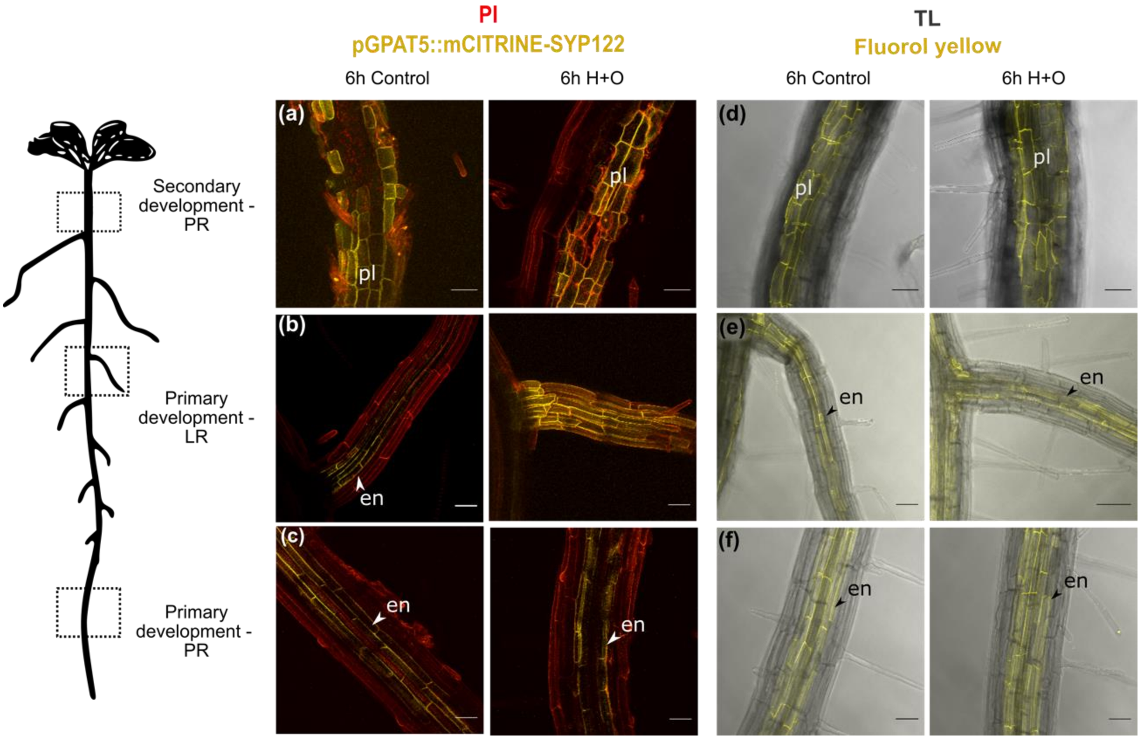

Figure S2 Effect of combined heat and osmotic stress on the suberization pattern of secondary (a, d) and primary development (b, c d, e) in lateral (LR) and primary root (PR) of Arabidopsis. Expression of suberin biosynthesis specific gene *GPAT5* in the promoter line pGPAT5::mCITRINE-SYP122 (yellow) was detected after 6h of mock and combined heat and osmotic stress treatments in different zones of root: (a) secondary developmental region, (b) lateral roots (LR) and in (a) young primary root. Propidium iodide (PI) was used to stain cell walls (red). Fluorol yellow staining (yellow) was used in col-0 Arabidopsis to detect suberin after 6h of mock and combined heat and osmotic stress treatments in the different zones of the root (d-f). The 3D maximum projection figures were obtained by confocal laser scanning with Z-stack images. Scale bar: 50 µm

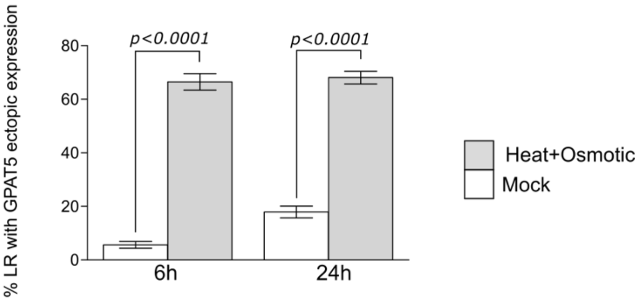

Figure S3 Percentage of lateral roots (LR) with ectopic pGPAT5::mCITRINE-SYP122 (*GPAT5*) expression after 6h and 24h of combined heat and osmotic stress. A total of 20 plants were analysed for each situation. The percentage is relative to total number of emerged LR. Statistical significance between treatments for each timepoint was assessed using the unpaired t-test.

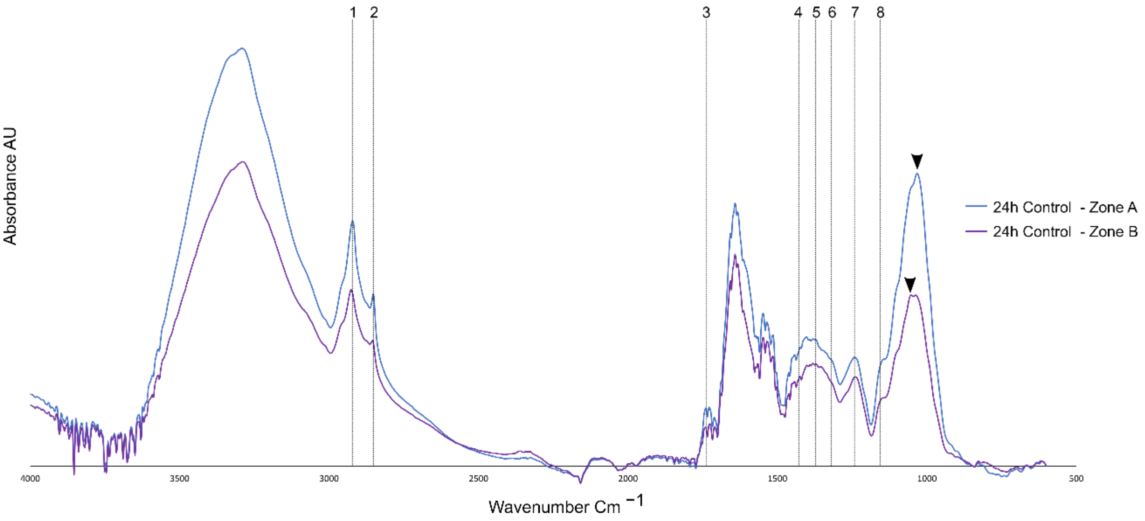

Figure S4 ATR-FTIR spectra of the Arabidopsis secondary and primary developmental zone of root, collected 24h under control conditions. Vertical dashed lines highlight major peaks assigned for suberin monomer groups: 1 and 2- long aliphatic chains of suberin (2921, 2852 cm^{-1}); 3, 7 and 8 – carboxyl and ester groups (1737, 1242, and 1158 cm^{-1}); 4 - phenolic compounds (1430 cm^{-1}); 5 and 6 - wax or suberin-like aliphatic components (1318 and 1372cm^{-1}). Arrowheads indicate different peaks detected between both samples.

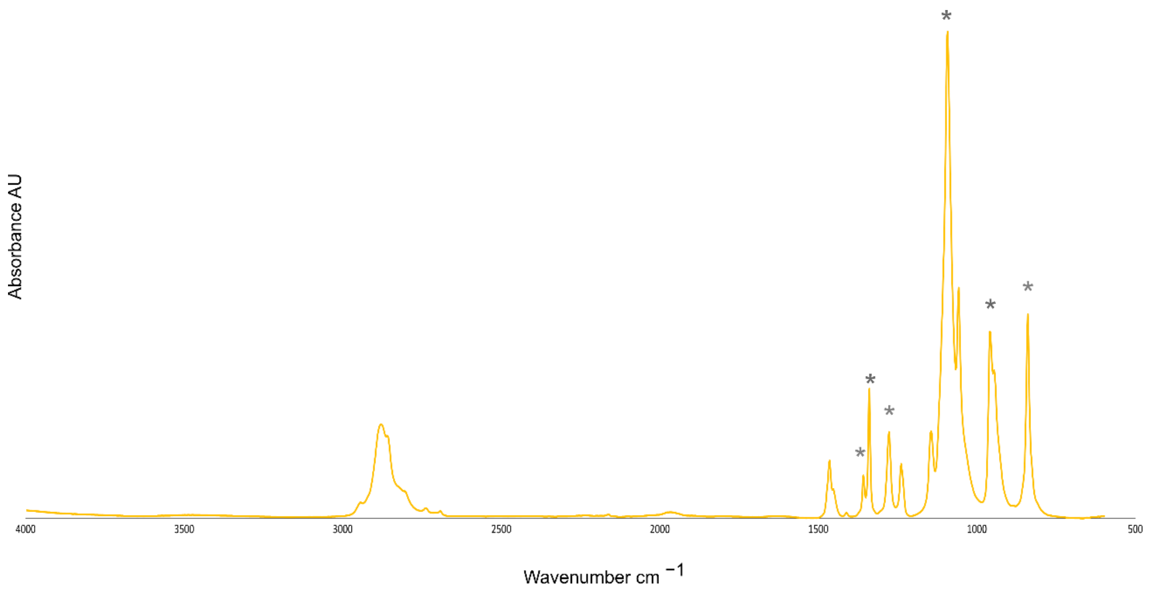

Figure S5 ATR-FTIR spectra of solid PEG 6000. Asterisks mark the possible contaminations pointed out in the ATR-FTIR spectra of Figure 5.

Table S1 Primers used in this work.

Gene ID	Primer name	Sequence
AT2G36100 (*CASP1*)	AtCASP1_qPCR1_Fw	CGAAGAAGAAGGGCTTTGTG
	AtCASP1_qPCR1_Rv	GCTTGGAACTGGAGGAACTG
AT5G25760 (*UBC21*)	AtUBC10_qPCR1_Fw	CTTGGACGCTTCAGTCTGTG
	AtUBC10_qPCR1_Rv	GGCGAGGCGTGTATACATTT
AT5G13580 (*ABCG6*)	AtABCG6_qPCR1_Fw	ATGAACCAACTTCGGGTCTG
	AtABCG6_qPCR1_Rv	CGGGACAAGAAGAGAAGACG
AT3G11430 (*GPAT5*)	AtGPAT5_qPCR1_Fw	GGGTTTGAGTGCACCAACTT
	AtGPAT5_qPCR1_Rv	GGAGACAAGGCTCGAAAGTG
AT5G41040 (*ASFT*)	AtASFT_qPCR1_Fw	GGTCAAACCTGAATCCGAGA
	AtASFT_qPCR1_Rv	CTTGGACTGCTTCCTCGTTC
AT5G48570 (*ROF2*)	AtROF2_qPCR_Fw	TCGACACGCAGAACCAGTTT
	AtROF2_qPCR_Rv	GATCTCCATTTCCTCGCCGA
AT5G25610 (*RD26*)	AtRD22_qPCR_Fw	GTAGGAGTCGGTAAAGGCGG
	AtRD22_qPCR_Rv	TAGGATCGTCGTGGAGCTGA
AT4G28110 (*MYB41*)	AtMYB41_qPCR_Fw	CAATAGCCGCTCGTCTACCA
	AtMYB41_qPCR_Rv	TCAAGGCGTGGAGAATGAGT

Table S2 Summary of main peaks obtained by ATR-FTIR that are assignment to cell wall components.

Wavelength (cm^{-1})	Assigned functional groups	Assigned functional group chemical structure	Assigned cell wall component	Reference
2921and 2852	Long aliphatic chains	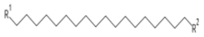	Suberin long chain aliphatic components	[1–3]
1737, 1242 and 1158	Carboxyl and ester groups	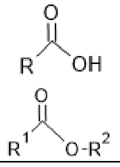	Carboxyl groups of the suberin fatty acids, ester groups of the suberin ester links	
1430	Phenolic compounds	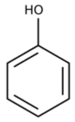	Suberin aromatic components	
1318 and 1372	Aliphatic components	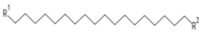	wax or suberin-like aliphatic components	
1032	Primary alcohols	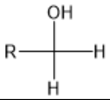	Polysaccharides	[4,5]
1055	Secondary alcohols	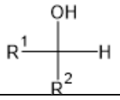	Polysaccharides	[4,5]
1407 and 1097	Galacturonic acid	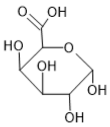	Pectin	[6,7]
2889	Glucose	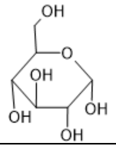	Cellulose	[8]

References

1. Zeier, J.; Schreiber, L. Fourier transform infrared-spectroscopic characterisation of isolated endodermal cell walls from plant roots: Chemical nature in relation to anatomical development. *Planta* **1999**, *209*, 537–542, doi:10.1007/s004250050758.

2. Ferreira, R.; Garcia, H.; Sousa, A.F.; Petkovic, M.; Lamosa, P.; Freire, C.S.R.; Silvestre, A.J.D.; Rebelo, L.P.N.; Pereira, C.S. Suberin isolation from cork using ionic liquids: characterisation of ensuing products. *New J. Chem.* **2012**, *36*, 2014, doi:10.1039/c2nj40433h.

3. Garcia, H.; Ferreira, R.; Martins, C.; Sousa, A.F.; Freire, C.S.R.; Silvestre, A.J.D.; Kunz, W.; Rebelo, L.P.N.; Silva Pereira, C. Ex situ reconstitution of the plant biopolyester suberin as a film. *Biomacromolecules* **2014**, *15*, 1806–1813, doi:10.1021/bm500201s.

4. Maréchal, Y.; Chanzy, H. The hydrogen bond network in I(β) cellulose as observed by infrared spectrometry. *J. Mol. Struct.* **2000**, *523*, 183–196, doi:10.1016/S0022-2860(99)00389-0.

5. Fahey, L.M.; Nieuwoudt, M.K.; Harris, P.J. Predicting the cell-wall compositions of Pinus radiata (radiata pine) wood using ATR and transmission FTIR spectroscopies. *Cellulose* **2017**, *24*, 5275–5293, doi:10.1007/s10570-017-

1506-4.

6. Alonso-Simón, A.; García-Angulo, P.; Mélida, H.; Encina, A.; Álvarez, J.M.; Acebes, J.L. The use of FTIR spectroscopy to monitor modifications in plant cell wall architecture caused by cellulose biosynthesis inhibitors. *Plant Signal. Behav.* **2011**, *6*, 1104–1110, doi:10.4161/psb.6.8.15793.

7. Szymanska-Chargot, M.; Zdunek, A. Use of FT-IR Spectra and PCA to the Bulk Characterization of Cell Wall Residues of Fruits and Vegetables Along a Fraction Process. *Food Biophys.* **2013**, *8*, 29–42, doi:10.1007/s11483-012-9279-7.

8. Tran, T.N.; Paul, U.; Heredia-Guerrero, J.A.; Liakos, I.; Marras, S.; Scarpellini, A.; Ayadi, F.; Athanassiou, A.; Bayer, I.S. Transparent and flexible amorphous cellulose-acrylic hybrids. *Chem. Eng. J.* **2016**, *287*, 196–204, doi:10.1016/j.cej.2015.10.114.
